# Systematic reviews and meta-analysis of preclinical studies: why perform them and how to appraise them critically

**DOI:** 10.1038/jcbfm.2014.28

**Published:** 2014-02-19

**Authors:** Emily S Sena, Gillian L Currie, Sarah K McCann, Malcolm R Macleod, David W Howells

**Affiliations:** 1 Department of Clinical Neurosciences, University of Edinburgh, Edinburgh, UK; 2 Stroke Division, Florey Institute of Neuroscience and Mental Health, Melbourne, Victoria, Australia

**Keywords:** acute stroke, animal models, basic science, Biostatistics, experimental

## Abstract

The use of systematic review and meta-analysis of preclinical studies has become more common, including those of studies describing the modeling of cerebrovascular diseases. Empirical evidence suggests that too many preclinical experiments lack methodological rigor, and this leads to inflated treatment effects. The aim of this review is to describe the concepts of systematic review and meta-analysis and consider how these tools may be used to provide empirical evidence to spur the field to improve the rigor of the conduct and reporting of preclinical research akin to their use in improving the conduct and reporting of randomized controlled trials in clinical research. As with other research domains, systematic reviews are subject to bias. Therefore, we have also suggested guidance for their conduct, reporting, and critical appraisal.

## Introduction

Animal models are invaluable tools for enriching our understanding of the mechanisms and etiology of human diseases. The number of preclinical experiments performed each year continues to increase and our understanding of disease mechanisms is improving, but the number of novel interventions reaching the clinic to treat cerebrovascular diseases continues to fall. It is clear that there are limitations to the translational paradigm as it currently exists. It is also clear from the sheer volume of preclinical research that structured methods are required to make objective sense of the available data. Systematic review and meta-analysis are useful tools which can address some, but not all, of the challenges of translational stroke research. They provide a less biased summary of research findings and allow judgement of both the range of available evidence (and hence the external validity) and the likelihood that conclusions are at risk of bias (the internal validity).

Systematic review sets out to use a structured process to identify all data relevant to a specific research question. This may be followed by meta-analysis, a statistical process that provides a summary estimate of the outcomes from a group of studies, and allows these outcomes from different groups of studies to be compared. Although the first meta-analysis was performed in 1904 by Karl Pearson, it was only in 1976 that Gene Glass coined the term ‘meta-analysis' to refer to this statistical pooling to allow the integration of findings. He suggests that meta-analysis was created out of the need to extract useful information from the cryptic records of inferential data analyses in the abbreviated reports of research in journals and other printed sources.^1^ Meta-analysis is now used in many fields of research including psychology, criminology, and education. Its use in clinical medicine is routine, and the Cochrane Collaboration has been instrumental in establishing the framework for evidence-based healthcare to guide clinical practice and healthcare policy. However, in preclinical research, the use of systematic review and meta-analysis is relatively novel.

Glass considers necessity was the mother of invention where meta-analysis is concerned; if it had not happened in the early 1970s, it was sure to happen soon after. We suggest that the same holds for meta-analysis in preclinical stroke research. In the early years of the millennium, the dogma had developed that ‘everything works in animals, but nothing works in humans'. In 2006, O'Collins *et al*^2^ published a review reporting that of more than 500 interventions that were reported to be efficacious in animal models of stroke, only thrombolysis with rtPA had been shown to be effective in stroke patients. A search for *in vivo* animal stroke studies published in the last 10 years yields more than 5,700 articles, but still no new therapies to treat acute stroke have been developed. If the purpose of preclinical cerebrovascular research was to develop new treatments for human stroke then clearly there were substantial problems. The complexities of translational research led our group, and others, to adapt the techniques of meta-analysis, at that time largely restricted to clinical research, to the preclinical domain, in an attempt to provide empirical evidence for weaknesses in the prevailing translational paradigm, evidence which might guide improvements in the translational process.

In this narrative review, we aim to explain the concepts of systematic review and meta-analysis; to describe how their use changed clinical medicine; and to explore the contribution they might make to preclinical research. We also describe some of the elements to consider in the critical appraisal of systematic reviews and meta-analyses of preclinical research.

## The concept

In any type of review, there are two fundamental steps that are taken; identify the studies that are relevant to your research questions and then synthesize these identified data to reach conclusions. The benefit of narrative reviews is that they include a broad overview of relevant information, perhaps interpreted by an experienced author tempered by years of practical knowledge of the field; in many cases, they are highly useful. Unfortunately, they also have limitations. The selection methods used to identify studies that contribute to the review are often not transparent. The credence given to individual studies is inherently subjective and often unclear. Often, the reviewer themselves may be unable to articulate the processes through which they reached the conclusions presented. Systematic reviews are not bias free, but the transparency of the methods used are designed to reduce bias. In a systematic review, the researcher is required to outline aims, objectives, and methodology. The principle is that an independent researcher could perform the same identification process and yield the same data set. As is often the case, in the interpretation of primary research studies, the conclusions drawn from a meta-analysis may differ from one reviewer to the next. However, the transparency and objectivity of the techniques used provide a framework for these discussions.

The data synthesis process in meta-analysis has a number of steps once the individual studies have been identified. First, the effect sizes of individual studies are determined. This is often a treatment effect that is a measure of the difference between control and treatment groups. Effect sizes are not limited to effects of drugs, but may represent a relationship between any two variables. Second, the precision of an effect size is determined by its standard error. The broad aim is to calculate an average effect size across the studies, termed the summary estimate of effect. Third, because some effect sizes are more precise than others, this averaging process is often weighted so that more precise studies are given more weight in a meta-analysis than less precise ones. Finally, the differences between the component effect sizes—the heterogeneity–are assessed. We expect variation of effect sizes to occur due both to random error and to real differences in experimental design. In our view, it is this exploration of sources of heterogeneity—the identification of those aspects of experimental design that cause exaggerations or underestimations in treatment effects, or those aspects of drug delivery that give maximum efficacy—which is the true strength of meta-analysis. However, because we are simply observing a “cohort” of experiments, rather than testing the impact of these influences experimentally, findings from meta-analysis should be considered hypothesis generating rather than confirmatory.

## In clinical research

Although systematic reviews and meta-analyses are now routinely used by medical researchers to inform practice and policy, they also had a pivotal role in providing empirical evidence of the impact of bias in the conduct of controlled clinical trials. Chalmers and others provided evidence that studies that do not adequately mask treatment allocation are associated with bias and inflated treatment effects.^3, 4^ In the hierarchy of evidence randomized controlled trials are now considered the gold standard in clinical trial design. Conceptually, the ability of randomization to account for systematic differences in factors, known or unknown, between groups that may affect outcome is apparent; as is ensuring that preconceived views of patients and clinicians do not bias the assessment of outcomes.^5^ But it required empirical evidence to revolutionize clinical research and to convince trialists of the importance of methodological rigor in both the conduct and reporting of their studies.^6^

## What could meta-analysis do for preclinical research?

Across the modeling of a number of cerebrovascular diseases, there is a considerable volume of often conflicting data. Systematic review and meta-analysis can be used to describe which interventions have been tested in models of disease, to provide an indication of the attrition rate of interventions (i.e., the number of interventions not progressing to clinical trial), and to describe the range of conditions under which efficacy has been tested. Furthermore, pooling data using meta-analysis can be used to assess both the overall efficacy of an intervention and the impact of factors relating to internal and external validity, giving valuable insights into the causes of translational successes and failures.

In recent years we, and others, have presented empirical evidence that suggests that the usefulness of data from experiments testing drug efficacy in animal models of various neurologic diseases may be substantially impaired by limited methodological quality, limited generalizability, and by significant publication bias.^7, 8, 9, 10^

### Methodological Quality

In an experiment, the credibility of the inferred causal relationship between treatment and outcome is dependent upon on the statistical power and internal validity.

Preclinical animal research is confounded by pressures to reduce the number of animals used because of concerns about cost, time, ethics, and practicalities of disease modeling that might lead to studies either being underpowered or of unknown power. Determining the required sample size to answer a research question is crucial. Too small and the results are imprecise and lack statistical power. Too large and unnecessary costs are incurred. *A priori* sample size calculations also provide assurance that animals are not added to a study incrementally in response to (unreported) interim analyses.

The internal validity of an experiment ensures that the changes observed in outcomes are due to an induced change in one or more of the independent variables rather than some other confounding factor. The internal validity of an experiment may be threatened by a range of biases. These include, but are not limited to, selection bias, performance bias, and detection bias. Selection bias occurs when there are systematic differences between study groups at the start of an experiment. Performance bias occurs when systematic differences occur in how the groups are handled during a study and detection bias occurs when systematic differences occur between groups in how outcomes are ascertained, diagnosed, or verified. Measures to reduce the impact of these biases include randomization, allocation concealment and masked assessment of outcome.

Systematic review and meta-analysis of experimental studies covering a range of neurologic disorders have provided evidence that few studies take measures to reduce bias or perform formal power calculations to determine sample size (Table 1).

**Figure 1:**
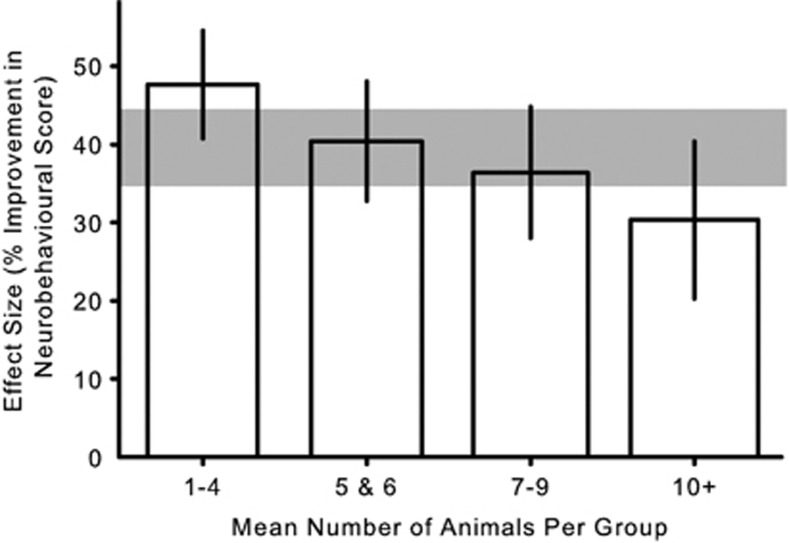
The effect of the mean sample size on the estimate of effect size for neurobehavioural score in models of encephalomyelitis. The horizontal gray bar represents the 95% confidence limits for the summary estimate of effect. The vertical error bars represent the 95% confidence intervals for the individual estimate. The widths of the bar represent the log of the number of animals contributing to that comparison.^11^

Meta-analysis can be used to assess the impact of methodological quality on reported outcomes. Unfortunately, sample size calculations are so seldom reported that it has not been possible to assess whether performing a sample size calculation influences outcome. However, in animal models of experimental autoimmune encephalomyelitis, we showed that reported efficacy was largest in the smallest studies^11^ (Figure 1).

Fortunately, we have been able to generate empirical evidence describing the impact of reporting of measures to reduce bias on outcome. In a meta-analysis of therapeutic hypothermia in experimental stroke, we observed treatment effects were 10% larger in non-randomized studies and 8% larger in unmasked studies than those that did take these measures to reduce bias.^12^ Similarly, in a meta-analysis of NXY-059 in experimental stroke, NXY-059 was reported to be 30% more effective in studies that were not randomized or masked than in studies that reported randomization and blinding.^13^

### External and Construct Validity

If preclinical models of disease are to inform human health, experiments require external validity and the models used require construct validity; both types of validity relate to the generalizability of a study. External validity refers to the ability to generalize the findings to different measures, settings, and times. Construct validity refers to adequate representation of theoretical constructs, in disease modeling this may be threatened where only specific characteristics of a complex disease are modeled. Aspects of both these validity types are clearly disease specific. For example, in the modeling of ischemic stroke, associated co-morbidities, age of animals, and the time to treatment are important factors. Using systematic review, we have identified that the majority of preclinical stroke studies use young male normotensive rats. Furthermore, meta-analysis has also provided evidence of no detectable effect of either tissue plasminogen activator or NXY-059 in hypertensive animals,^13, 14^ which would appear to be concordant with results in humans.

The most common recommendations from preclinical research guidelines to improve external validity are that experiments should be replicated in different models of the same disease, in different species and that findings should be replicated independently.^15^ Others recommend that the time that treatment is started after disease/injury induction should be realistic in terms of what is possible in the clinic.^16^ The most common recommendations to improve construct validity include characterization of disease phenotype in the animal model before experimentation, matching the model to the human disease and matching outcome measures to the clinical setting.^15^

### Reporting Bias

The validity of a systematic review may be limited by reporting biases of the component studies. It has long been recognized that neutral studies often remain unpublished or take longer to get published than those reporting statistically significant results. They are also more likely to be published in journals of low impact or in languages other than English.^17^ Such work is less likely to be identified in narrative and even in systematic review, and such publication bias can lead to the overstatement of summary effects in meta-analysis. Published meta-analyses now routinely report the presence or lack of publication bias in their reviews.

**Figure 2:**
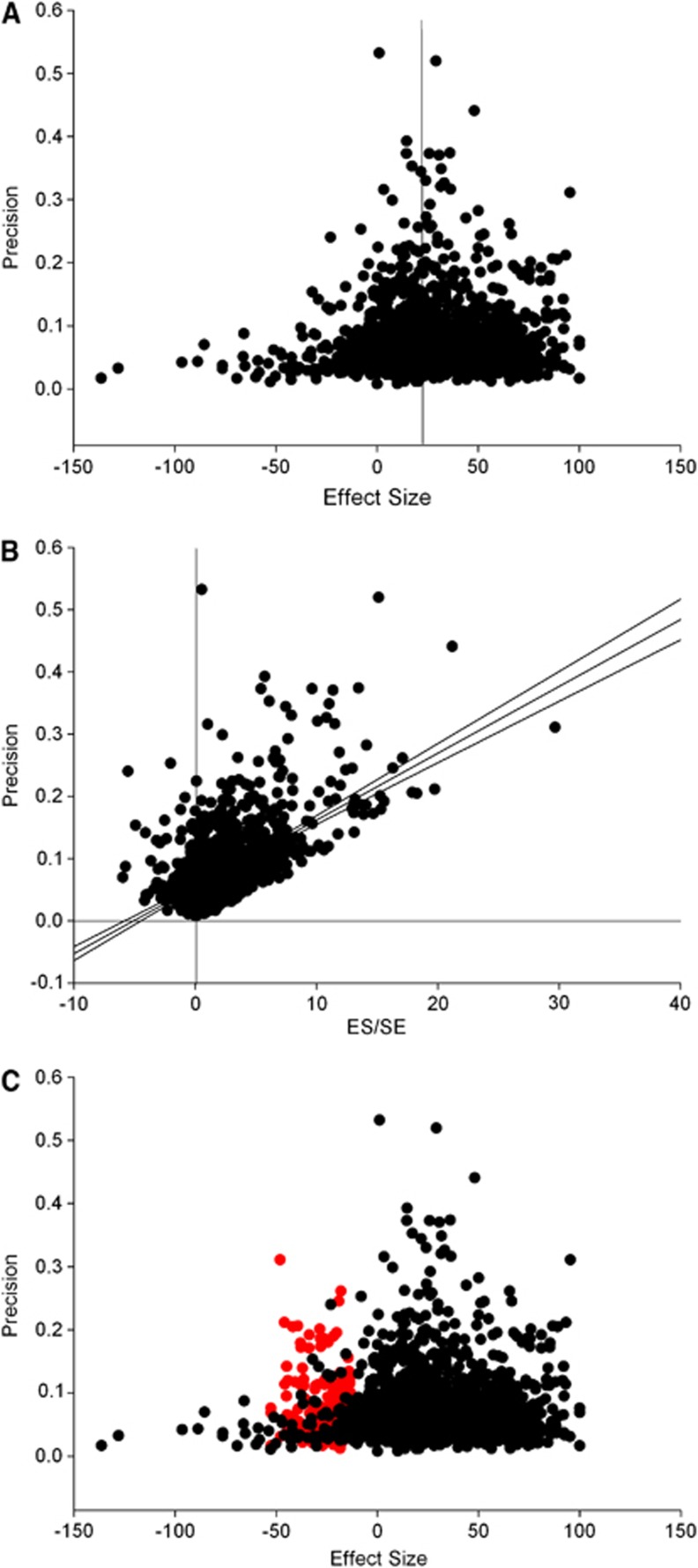
Publication bias. Plots describing (**A**) funnel plot, (**B**) Egger regression, and (**C**) trim-and-fill.^9^

Data from meta-analyses of 525 unique publications and 16 interventions tested in models of experimental stroke were combined and imprecise study effects consistent with publication bias were seen in funnel plot asymmetry and confirmed with Egger Regression (Figures 2A and 2B). Using a meta-analytical technique known as trim-and-fill that imputes theoretical missing studies, the overall efficacy was significantly reduced from 30.1% (28.7 to 31.6%) to 23.3% (21.7 to 24.9%), a relative overstatement in efficacy of 31%. Two hundred studies were deemed to be missing and were ‘filled' into the data set (Figure 2C). Furthermore, only 2% of publications reported no significant treatment effects.^9^

Other reporting biases that are less commonly assessed in systematic reviews include selective outcome reporting and selective analysis reporting. These biases may occur where many outcome measures are assessed or many statistical analyses are performed but only the ‘best' results are presented. This in turn leads to a body of evidence with an inflated proportion of statistically significant results.^18^ In a review of 160 meta-analyses including 4,445 experiments from the modeling of six neurologic disorders (Alzheimer's, encephalomyelitis, focal ischemia, intracerebral hemorrhage, Parkinson's disease, and spinal cord injury) we assessed, using the Excess Significance Test, whether too many of the individual studies in the meta-analyses reported statistically significant results.^10^ We expected 21% of the results to be significant but observed 39% significant results, suggesting the presence of bias in this data set. These issues may, in part, be addressed by the use of published protocols.

## How to appraise critically a systematic review and meta-analysis of preclinical studies

In a systematic review of systematic reviews of preclinical studies, Mignini and Khan found that 30% specified a testable hypothesis, 27% performed a literature search without language restrictions, 17% assessed for the presence of publication bias, half assessed study validity, and 2% investigated sources of heterogeneity.^19^ As with any type of research, systematic reviews and meta-analyses are susceptible to bias, and it is only through clear reporting of what was done that it is possible to assess this risk of bias.

As systematic reviews and meta-analyses of preclinical research become more common, a number of different approaches have been used and readers—and reviewers—need to be able to assess whether the methodologies used are sound and the interpretation is valid. However, we are not aware of any study that provides empirical evidence of the presence or magnitude of the risk of bias associated with different aspects of the conduct or reporting of systematic reviews and meta-analyses of data from *in vivo* experiments.

Without such data, guidelines have less validity, but in a developing field, it is, we believe, reasonable to make some recommendations, based in part on our experience in conducting such reviews and in part on guidelines in other, related fields. In Table 2, we make some recommendations for reporting systematic reviews and meta-analyses of animal studies using key elements of the guidelines proposed by Peters *et al*^20^ that are akin to the PRISMA guidelines for the reporting of systematic reviews and meta-analyses of healthcare interventions in human clinical studies.^21^ We suggest the following steps should be considered in the critical appraisal of a systematic review and meta-analysis (Table 3; adapted from Garg *et al*^22^).

### Does the Study Follow a Pre-Specified Protocol?

If a protocol exists, the manuscript should provide a reference to where it might be found.

A pre-specified written protocol is likely to improve the standard of a systematic review and meta-analysis by reducing the risk of ‘data dredging' and *post hoc* revisions of study aims. Making the protocol publicly available to the research community also allows reviewers to obtain feedback on drafts through peer review. It is often the case that protocols evolve during the course of the review in light of clearer understanding of the research field and the data available; if such iterations occur, the changes should be justified and the date of these changes should be given in a revised protocol. The protocol should clearly define the research question, objectives, inclusion criteria, search strategy, data collection processes, and data analysis plan. A protocol should allow readers to judge whether the final study did indeed follow a pre-specified plan.

### Was the Research Question Focused and Clearly Defined?

The manuscript should provide a clear statement of the research question that is addressed

As with any study, the research question of a systematic review and meta-analysis needs to be focussed and clearly defined. It also needs to be appropriate to the type of study being performed, and its answer meaningful to the field.

### Are the Inclusion Criteria Appropriate?

The manuscript should define clear inclusion and exclusion criteria relating to the identification of relevant publications.

The reader should be confident that the eligibility criteria for inclusion in a systematic review are not biased and are appropriate to the research questions being asked. The scope of the inclusion criteria determines the validity of the conclusions drawn. There has been a debate on how broad or narrow the selection process should be;^23^ some argue that only studies that meet a high standard of methodological quality should be included^24^ while others argue that meta-analyses should deal with the good, bad, and indifferent of included studies.^1^ There is no correct approach but the degree of caution with which the results are interpreted and conclusions drawn should take this into account. Some reviews are restricted to studies published in English, we suspect because of the ease of data abstraction. Language bias occurs if studies performed in non-English speaking countries are more likely to publish their statistically significant results in English language journals and non-significant results in non-English language journals.^25^ The impact of language bias on systematic reviews of preclinical data is yet to be determined. It may not be always practical to translate all non-English studies, but the reviewers should at least identify and report how many studies were excluded for reason of language. This is usually possible as titles and abstracts are often translated even if the full papers are not.

### How Comprehensive was the Search Strategy?

The manuscript should define the search strategy, including the search terms used, the databases searched, and the date(s) of the search(es).

The number of investigators screening publications for inclusion, and the method for dealing with inconsistencies, should be described.

Identifying relevant studies for a systematic review can be arduous. Biomedical journals are the most common source of relevant data and these are identified via the searching of bibliographic databases. A range of bibliographic databases should be searched as no database has complete coverage of all health-related literature. The core databases used are Medline and Embase; Medline is often searched via PubMed, but other search services may be used. Embase indexes more European and Asian journals and has roughly a 60% overlap with Medline. There are other sources of data that may be appropriate, including other specialized databases, conference proceedings, personal communications, and books.

The search strategy used needs to be comprehensive enough to identify most relevant studies. The screening of identified studies for inclusion is susceptible to random error and may be subjective. For this reason, we advise that two independent reviewers screen studies for inclusion, and the number of reviewers should be reported in the manuscript.

A comprehensive search reduces the possibility of publication bias in a review. Publication bias may be present because of an incomplete search of the literature or because the studies themselves are not in the public domain to be identified in the search process. There are various techniques for assessing for the presence and impact of publication bias in a meta-analysis^17^ that reviewers should consider.

### Was the Data Abstraction from Each Study Appropriate?

The choice of data (times, outcome measures) to be extracted from each publication should be defined.

The number of investigators extracting data from publications, and the method for dealing with inconsistencies, should be described.

The reviewers should be rigorous and their methods reproducible in extracting data for included studies. Ideally, this process should also be performed by two independent reviewers. The assessment of methodological quality of included studies should be reported. Understanding the rigor and validity of included studies is important in the interpretation of the conclusions drawn.

### Were the Data Pooled Appropriately?

The manuscript should identify a primary outcome variable or the statistical limits to any subdivision of the data.

The manuscript should describe the method of pooling of data and provide summary estimates, estimates of uncertainty (e.g. 95% confidence intervals), and a measure of heterogeneity (e.g. Q, I^2^).

The statistical pooling of aggregate data in a meta-analysis gives greater weight to more precise studies. The assumption of this pooling may be performed under a ‘fixed' or ‘random' effects model. More detailed discussion of the merits of each model in the pooling of preclinical data can be found elsewhere^26^ but on the whole, given the heterogeneity often observed in preclinical studies, random effects meta-analysis is more appropriate. Formal assessment of heterogeneity should be performed. Attempts to stratify data to account for, or explain, some of the heterogeneity are useful.

## Summary

Recent years have seen improvements in the conduct and reporting of clinical trial design after the publication of the CONSORT statement.^27^ Analogous to this, the ARRIVE guidelines^28^ and Landis paper^29^ hope to promote the same improvements in animal experiments.

Systematic review and meta-analysis have provided empirical evidence that too many preclinical experiments lack methodological rigor, and this leads to inflated treatment effects. There is of course no guarantee that improvements in the validity of preclinical animal studies and reduced publication bias will improve the translational hit of interventions from bench to bedside. However, we hope that the mounting empirical evidence drives the preclinical research community toward improved rigor. Despite some very reasonable concerns about the novelty of the methodological approach and the difficulty of confirming these hypotheses experimentally, findings from meta-analyses cover such a wide range of disease models and have been reported by so many different research groups that it is highly unlikely that these conclusions do not reflect a real and present problem with the use of animal models. As consumers of systematic reviews and meta-analyses of preclinical research, it is important that we are able to discern, as we do for primary research studies, the rigor with which the meta-analyses are performed and reported.

We hope that this empirical evidence of bias, akin to that reported in clinical research more than 30 years ago, spurs a similar change in the way we conduct and report preclinical research. Whether this leads to improved translation we are yet to see. However, what we will observe is an improvement in the conduct and reporting of preclinical research that reasonably can only be of benefit to this troubled field of research.

## Figures and Tables

**Table 1:** Number and percentages of studies across the modeling of different neurologic diseases reporting measures to reduce the risk of bias

	*Number of publications*	*Masked assessment of outcome (%)*	*Random allocation to group (%)*	*Allocation concealment (%)*	*Sample size calculation (%)*
Alzheimer's disease^30^	428	95 (22)	67 (16)	NA	0 (0)
Multiple sclerosis^11^	1,117	178 (16)	106 (9)	NA	2 (<1)
Parkinson's disease^31^	252	38 (15)	40 (16)	NA	1 (<1)
Intracerebral hemorrhage^32^	88	43 (49)	27 (31)	7 (8)	0 (0)
					
*Focal ischemia*					
NXY 059^13^	9	4 (44)	3 (33)	5 (56)	2 (22)
Hypothermia^12^	101	38 (38)	36 (36)	4 (4)	0 (0)
Erythropoietin^33^	19	8 (42)	7 (37)	4 (21)	0 (0)
Tirilazad^34^	18	13 (72)	12 (67)	1 (6)	0 (0)
tPA^14^	113	24 (21)	42 (37)	23 (20)	8 (7)

NA, not applicable; tPA, tissue plasminogen activator.

**Table 2:** Guidelines for reporting systematic reviews and meta-analyses of animal studies

Title	Identify the report as a systematic review and/or meta-analysis of animal experiments.
Abstract	Provide a structured abstract covering the following: objectives, data sources, review methods, results, and conclusion.
Introduction	Clearly defined and focussed research question.
	
*Methods*	
** **Protocol	Indicate if a protocol exists and where it can be found (i.e., web address).
** **Searching	Describe the information sources in detail, including keywords, search strategy, any restrictions, and special efforts to include all available data.
** **Selection	Describe the inclusion and exclusion criteria.
** **Validity and quality assessment	Describe the criteria and process used to assess validity.
** **Data abstraction	Describe the process or processes used (e.g., completed independently, in duplicate).
	Describe whether aggregate data or individual animal data are abstracted.
** **Study characteristics	Describe the study characteristics relevant to your research question.
** **Quantitative data synthesis	Describe the principal measures of effect, method of combining results, handling of missing data; how statistical heterogeneity was assessed; and any assessment of publication bias—all in enough detail to allow replication.
	
*Results*	
** **Flow chart	A meta-analysis profile summarizing study flow giving total number of experiments in the meta-analysis.
** **Study characteristics	Descriptive data for each experiment.
** **Quantitative data synthesis	Present simple summary results (e.g., forest plot); identify sources of heterogeneity, impact of study quality, and publication bias.
Discussion	Summarize the main findings; discuss limitations; provide general interpretation of the results in the context of other findings, and implications for future research.
Funding	Describe sources of funding for the review and other support. The role of funders should be presented.
Conflict of interest	Any potential conflict of interests should be reported.

**Table 3:** Points to consider in the critical appraisal of systematic reviews and meta-analyses of animal studies


1. Does the study follow a pre-specified protocol?
2. Was the research question focused and clearly defined?
3. Are the inclusion criteria appropriate?
4. How comprehensive was the search strategy?
5. Was the data abstraction from each study appropriate?
6. Were the data pooled appropriately?
